# Endemic Babesiosis in Another Eastern State: New Jersey

**DOI:** 10.3201/eid0902.020271

**Published:** 2003-02

**Authors:** Barbara L. Herwaldt, Paul C. McGovern, Michal P. Gerwel, Rachael M. Easton, Rob Roy MacGregor

**Affiliations:** * Centers for Disease Control and Prevention, Atlanta, Georgia, USA; † University of Pennsylvania Health System, Philadelphia, Pennsylvania, USA; ‡ New Jersey Department of Health and Senior Services, Trenton, New Jersey, USA

**Keywords:** New Jersey, babesiosis, *Babesia microti*, tick, *Ixodes scapularis*, surveillance, research

## Abstract

In the United States, most reported cases of babesiosis have been caused by *Babesia microti* and acquired in the northeast. Although three cases of babesiosis acquired in New Jersey were recently described by others, babesiosis has not been widely known to be endemic in New Jersey. We describe a case of babesiosis acquired in New Jersey in 1999 in an otherwise healthy 53-year-old woman who developed life-threatening disease. We also provide composite data on 40 cases of babesiosis acquired from 1993 through 2001 in New Jersey. The 40 cases include the one we describe, the three cases previously described, and 36 other cases reported to public health agencies. The 40 cases were acquired in eight (38.1%) of the 21 counties in the state. Babesiosis, a potentially serious zoonosis, is endemic in New Jersey and should be considered in the differential diagnosis of patients with fever and hemolytic anemia, particularly in the spring, summer, and early fall.

In the United States, most of the hundreds of reported cases of babesiosis have been caused by *Babesia microti*, a parasite of small mammals transmitted by *Ixodes scapularis* (deer ticks); these ticks also transmit *Borrelia burgdorferi* and *Anaplasma (Ehrlichia) phagocytophila*. Most reported cases of babesiosis have been acquired in the northeast, specifically in New York, Massachusetts, Connecticut, and Rhode Island. Another focus of *B. microti* infection is in Wisconsin and Minnesota (*1*).

Although three cases of babesiosis acquired in New Jersey in 1998 were described by Eskow et al. (*2*), babesiosis has not been widely known to be endemic in New Jersey. Of interest, the index case-patient who acquired *B. microti* infection in the northeast (on Nantucket Island in 1969) actually was hospitalized in New Jersey (*3*). We describe a case of babesiosis acquired in New Jersey in 1999 and provide composite data that included this case, the three cases previously reported by Eskow et al. (*2*), and 36 other cases acquired in New Jersey from 1993 through 2001. Our data strengthen the conclusion that babesiosis is endemic in New Jersey.

## Methods

### Case Detection and Definition

We learned of additional babesiosis cases because they were reported to the New Jersey Department of Health and Senior Services or because health-care providers contacted the Centers for Disease Control and Prevention (CDC) about the diagnosis or treatment of babesiosis. Although babesiosis is not a nationally notifiable disease, some states have made cases of babesiosis reportable. Cases became reportable in New Jersey in 1985; however, reporting was discontinued in 1990 because no cases had been reported. Reporting was reinstated in 1995, and 1997 was the first year in which cases were reported to the health department.

We defined a potential case of babesiosis as an infection occurring in a symptomatic person whose illness was consistent with babesiosis, most likely was acquired in New Jersey, and most likely resulted from a tick bite rather than a blood transfusion. In addition, supporting laboratory data had to be provided and include at least one of the following: identification by light microscopy of intraerythrocytic *Babesia* parasites in a peripheral blood smear, isolation of the parasite from a whole blood specimen (by inoculating hamsters [*Mesocricetus auratus*] intraperitoneally and examining blood smears obtained weekly by tail snip for up to 2 months), demonstration of *B. microti* DNA in a whole blood specimen by polymerase chain reaction (PCR) analysis at a reference laboratory, or demonstration of a *Babesia-*specific antibody titer of at least 1:256 with an indirect fluorescent antibody assay for total immunoglobulin (Ig) G. If only serologic data met the diagnostic criteria, the case was considered probable rather than confirmed.

### Case Report

**Figure 1:**
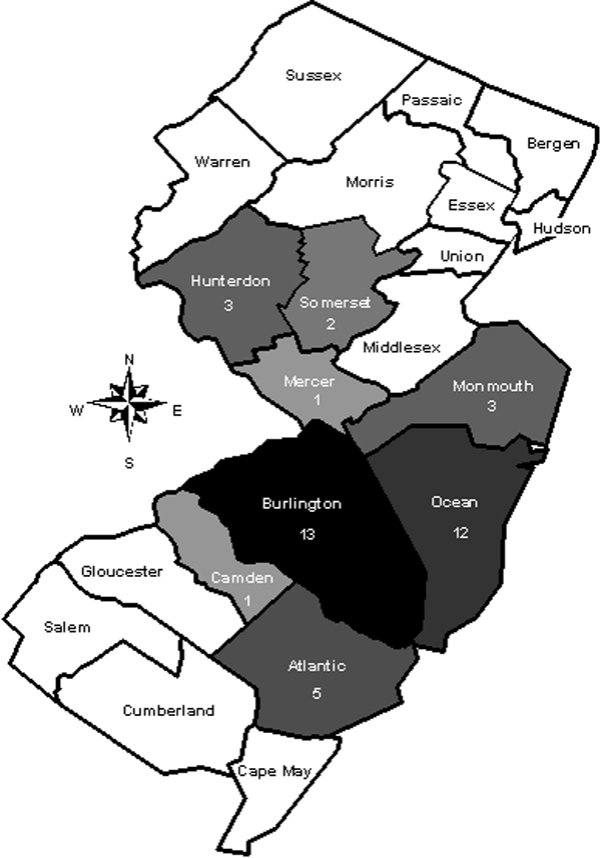
Map of New Jersey showing its 21 counties. The eight counties in which reported cases of babesiosis were acquired from 1993 through 2001 are shaded in gray (the darker the gray, the more cases). The number of cases reported per county is shown under the name of the county.

A previously healthy 53-year-old woman was admitted to a community hospital on June 24, 1999, because she had 1 week of fever (38.9°C–39.4°C), rigors, a nonproductive cough, an occipital headache, and increasing malaise. Three days before her hospitalization, she started therapy with cefuroxime axetil for presumed bronchitis but did not improve. She had a >50 pack-year history of smoking and drank two to three beers per day. She lived in Burlington County (Figure 1) in southcentral New Jersey and had not traveled outside the county recently. Although she did not recall recent exposure to deer ticks, she occasionally had seen deer in her backyard and she gardened frequently.

On admission to the hospital, she had a temperature of 39.2°C, a blood pressure level in the 80/60 mm Hg range, and otherwise unremarkable results on physical examination. She was anemic and thrombocytopenic, with elevated total bilirubin and lactate dehydrogenase values (Table). On the basis of a blood smear from June 24, which showed intraerythrocytic ring forms in approximately 5% of the erythrocytes on her peripheral blood smear, treatment for babesiosis was begun on June 25. The treatment included intravenous clindamycin, 900 mg three times a day, and 650 mg oral quinine three times a day; and she was transfused with two units of packed erythrocytes. Also on June 25, hypoxic respiratory failure developed, and she was intubated. A chest radiograph showed diffuse alveolar infiltrates, which were attributed to the adult respiratory distress syndrome (ARDS).

**Table:** Clinical data on selected dates for a patient who acquired babesiosis in New Jersey

Date	Temperature (^°^C)	Hematocrit (%)	Leukocyte count (10^9^/L)	Platelet count (10^9^/L)	Parasitemia level (%)	Creatinine level (mg/dL)^a^	Total bilirubin level (mg/dL)^a^	Lactate dehydrogenase level (U/L)^a^	Comments
June 24	39.2	25	4.6^b^	92	5.0	1.1	2.2^c^	646	Hospitalized
June 25	38.2	—	—	—	—	—	3.2	—	Antibabesial therapy started; intubated 2 units packed erythrocytes transfused
June 26	40.0	31	6.0	55	—	1.1	2.9	1,019	—
June 28	—	28	8.6	93	—	0.9	2.7^d^	—	Transferred to the Hospital of the University of Pennsylvania
June 29	39.2	27	9.0	99	0.3	1.9	—	5,147	—
June 30	38.3	22	7.9	109	—	2.8	1.5	4,047	2 units packed erythrocytes transfused
July 1	38.7	26	9.7	138	—	3.5	1.9	4,140	—
July 2	38.0	26	9.0	139	—	2.9	1.6	3,669	2 units packed erythrocytes transfused
July 4	38.7	29	14.7	144	—	2.2	2.2	2,864	Extubated
July 7	38.2	28	19.3	277	—	1.5	—	—	Developed nosocomial pneumonia
July 8	38.1	23	16.3	308	—	1.7	2.1	2,008	2 units packed erythrocytes transfused
July 9	38.3	31	11.7	300	0.0	1.8	—	—	Antibabesial therapy stopped
July 11	Afebrile	28	15.3	310	—	1.8	—	—	—
July 16	Afebrile	29	15.8	396	—	1.5	1.0	—	—
July 19	Afebrile	30	15.8	322	—	1.6	—	1,081	—
July 21	Afebrile	26	12.3	71	—	1.9	—	—	Developed heparin-associated thrombocytopenia
July 23	Afebrile	28	12.4	50	—	—	—	—	Sent home

^a^Normal ranges for community hospital (June 24–June 27): creatinine level, 0.6–1.3 mg/dL; total bilirubin level, 0.2–1.0 mg/dL; indirect bilirubin level, 0.2–1.2 mg/dL; lactate dehydrogenase level, 91–180 U/L. Normal ranges for the Hospital of the University of Pennsylvania: creatinine level, 0.6–1.0 mg/dL; total bilirubin level, 0.0–1.2 mg/dL; indirect bilirubin level, 0.0-1.2 mg/dL; lactate dehydrogenase level, 313–618 U/L.

^b^25% segmented neutrophils, 33% band forms, 22% lymphocytes, 2% atypical lymphocytes, 8% monocytes.

^c^Indirect bilirubin level, 1.4 mg/dL.

^d^Indirect bilirubin level, 2.1 mg/dL; serum haptoglobin level, <38 mg/dL (normal range, 60–160 mg/dL); results of direct and indirect Coombs test, negative.

On June 28, she was transferred to the Hospital of the University of Pennsylvania. When admitted, her blood pressure was 84/52 mm Hg, despite therapy with dopamine. She continued therapy for babesiosis for a total of 15 days (the dose of clindamycin was decreased to 600 mg three times a day on June 28). Although the level of parasitemia had decreased to 0.3% by June 29, she had ongoing hemolysis and received six more units of packed erythrocytes during her hospital stay (Table). No parasites were noted on a blood smear on July 9, the last day of antibabesial therapy. She had been successfully weaned from inotropic blood-pressure support on July 2 and underwent extubation on July 4.

Additional laboratory testing at CDC provided further evidence that she was infected with *B. microti*. Serum specimens assayed in parallel, in serial fourfold dilutions, by indirect fluorescent antibody testing for antibody to *B. microti* (*4*), had titers of 1:1,024 (June 30, 1999) and 1:16 (July 16, 2000). In addition, PCR analysis of whole blood from June 30, 1999, by using *B. microti*-specific primers (*5*), confirmed she was infected with *B. microti*. Serologic testing performed at the Hospital of the University of Pennsylvania by enzyme immunoassay for antibody to *Borr. burgdorferi* was negative.

Complications during her hospitalization unrelated to babesiosis included nosocomial pneumonia, acute tubular necrosis from hypoperfusion, bilateral deep venous thromboses, pulmonary embolism, and thrombocytopenia temporally associated with the initiation of heparin therapy (Table). On July 23, after 30 days in the hospital, she was sent home. She was continuing to do well as of October 2002.

### Composite Data

The 40 cases in our analyses included the case described above, the three cases previously described by Eskow et al. (*2*), and 36 other cases. We did not include six other reported tick-borne cases that occurred in New Jersey residents, because the laboratory data did not meet our criteria or information about the probable state in which infection was acquired was not known or provided.

**Figure 2:**
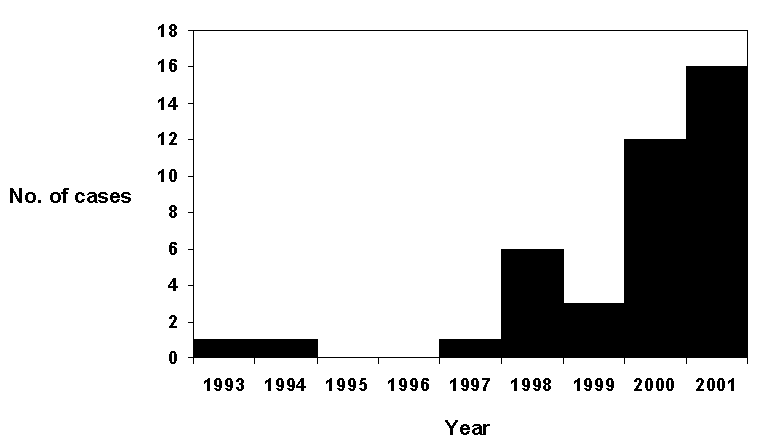
The number of reported cases of babesiosis acquired each year, 1993–2001.

The number of reported cases of babesiosis increased over time (Figure 2); 28 (70.0%) of the 40 cases occurred in 2000 or 2001. The 40 cases were acquired in eight (38.1%) of the state’s 21 counties (Figure 1). Burlington County, on the inner coastal plain, and Ocean County, on the outer coastal plain, which are neighboring counties in southcentral New Jersey, accounted for 25 (62.5%) of the 40 cases; these two counties are the 7th (Ocean) and 10th (Burlington) most populous counties in the state. None of the cases were acquired in the northernmost or southernmost counties of New Jersey.

Most of the cases were in elderly persons (median age, 67 years; range, 11–87 years). Over half of the cases (22 [55.0%]) were in male patients. The median date of diagnosis was July 20 (range, June 10–September 9; n=36). Two patients (5.0%) died: an 86-year-old man with multisystem organ failure and an 80-year-old man with ARDS. The patient whose case we described here also developed ARDS.

The following information about the patients was not collected systematically. However, three patients were reported to be asplenic, 18 to have recalled tick bites, 34 to have been hospitalized, and three to have had Lyme disease (no details available). Underlying conditions included HIV infection in one patient, who had a CD4 count of 50; diabetes in five patients; a history of breast or prostate cancer in three patients (no details available); and a condition that led to chemotherapy in one person (no details available).

Various types of laboratory tests were used to diagnose the 40 babesiosis cases. Not all patients were tested with the same methods; however, 34 patients had positive blood smears; for 27 of these patients, the positive smear was the only laboratory result that met our diagnostic criteria. All three cases reported by Eskow et al. were in patients who had negative blood smears (*2*). One of the two patients who had whole blood inoculated into hamsters had positive results (i.e., the hamsters became parasitemic). Four patients had positive PCR results from a reference laboratory; for one of these patients, these results were the only ones that met our diagnostic criteria. Twelve patients had serologic data that met our criteria; for four patients, these data were the only ones that met our diagnostic criteria. These cases were considered probable rather than confirmed. CDC confirmed the diagnosis of *Babesia* infection in 11 (27.5%) of the 40 cases; specimens from the other 29 case-patients were not sent to CDC.

## Discussion

Our report strengthens the evidence that New Jersey is one of the eastern states in which babesiosis is endemic. In addition, the risk for acquisition of infection is widely distributed in the state. Whether the fact that most of the reported cases occurred in southcentral and northcentral counties reflects the degree of endemicity of babesiosis in various areas of New Jersey is unknown.

The fact that babesiosis is endemic in New Jersey is not surprising, given that Lyme disease, the etiologic agent of which also is transmitted by *I. scapularis*, is highly endemic in New Jersey (*6*,*7*) and given the geographic proximity of New Jersey to areas in the northeast where babesiosis is highly endemic. In a 1996 study, of 100 *I. scapularis* ticks collected in Hunterdon County, New Jersey, 43 were infected with *Borr. burgdorferi*, 5 were infected with *B. microti*, and 2 were infected with both organisms (*8*).

The increase in reported cases of babesiosis, which began in 1998 (Figure 2) and escalated in 2000 and 2001, could indicate an increased risk of *B. microti* infection and illness. If true, possible reasons for the increased risk could include a growing abundance of local *I. scapularis* populations or the introduction of a more virulent strain of *B. microti* (*9*). However, the increased numbers of reported cases could simply represent an increased awareness of the disease and increased reporting. Even so, the 40 cases of babesiosis that we tallied probably represent only a fraction of the clinical cases of *B. microti* infection acquired in New Jersey from 1993 through 2001. Presumably, other symptomatic cases (as well as many more subclinical cases) occurred but were not diagnosed or reported. In fact, several other possible symptomatic cases were reported that we did not count because we received insufficient information. Also, as is commonly true for surveillance data, the amount and quality of the information provided to the health department and CDC about the cases varied widely; some of the information might have been inaccurate, and not all of the cases were confirmed by reference laboratories (e.g., not all of the blood smears that were reported as positive were reexamined by a reference laboratory).

The laboratory tests CDC offers for babesiosis, when indicated, include examination of blood smears, hamster inoculation, and PCR (*5*) for parasitologic diagnosis and an indirect fluorescent antibody assay for total immunoglobulin for serologic diagnosis (*4*). Using PCR for detection of DNA from *Babesia* spp. has not yet become a routine diagnostic method, and the analysis should be conducted by experienced reference laboratories.

Immunoblot testing for IgG and IgM is investigational. However, an immunoblot test for IgG performed well in a recent evaluation, with a sensitivity of 96% and a specificity of 99% (*10*). A positive serologic result for IgM (*11*) is insufficient for diagnosis without a positive result for IgG. If the IgM result is positive but the IgG result is negative, a follow-up specimen should be tested. If IgG seroconversion is not noted, the IgM result likely was a false positive. Future serologic testing might involve recombinant and synthetic antigens (*12*) rather than whole parasites or soluble antigens.

The case we described in detail demonstrates that babesiosis can be life threatening (*1*,*13*,*14*). In fact, two (5.0%) of the 40 case-patients died. In the patient we described, the following conditions developed: severe anemia, for which she was transfused with eight units of packed erythrocytes; hypotension that required inotropic support; ARDS, which has previously been reported (*13*–*17*); and various nosocomial complications. The fact that she was ill for approximately 1 week before therapy for babesiosis was initiated might have contributed to the severity of her illness. Fortunately, treatment for babesiosis was begun soon after she was hospitalized. Although she was treated successfully with clindamycin and quinine, a recent clinical trial indicated that the combination of azithromycin and atovaquone is also effective (*18*). However, patients with life-threatening babesiosis were excluded from the study. Severely ill patients, such as those with high levels of parasitemia, may benefit from exchange transfusion (*1*,*19*).

In summary, babesiosis, a potentially serious zoonosis, is endemic in New Jersey and should be considered in the differential diagnosis of patients with fever and hemolytic anemia, particularly in the spring, summer, and early fall.
